# Elevated CO_2_ reduces copper accumulation and toxicity in the diatom *Thalassiosira pseudonana*

**DOI:** 10.3389/fmicb.2022.1113388

**Published:** 2023-01-06

**Authors:** Dong Xu, Shujie Huang, Xiao Fan, Xiaowen Zhang, Yitao Wang, Wei Wang, John Beardall, Georgina Brennan, Naihao Ye

**Affiliations:** ^1^Yellow Sea Fisheries Research Institute, Chinese Academy of Fishery Sciences, Qingdao, China; ^2^Function Laboratory for Marine Fisheries Science and Food Production Processes, Qingdao National Laboratory for Marine Science and Technology, Qingdao, China; ^3^School of Biological Sciences, Monash University, Clayton, VIC, Australia; ^4^Institute of Marine Sciences, ICM-CSIC, Barcelona, Spain

**Keywords:** ocean acidification, copper accumulation, copper toxicity, adaptation, *Thalassiosira pseudonana*

## Abstract

The projected ocean acidification (OA) associated with increasing atmospheric CO_2_ alters seawater chemistry and hence the bio-toxicity of metal ions. However, it is still unclear how OA might affect the long-term resilience of globally important marine microalgae to anthropogenic metal stress. To explore the effect of increasing *p*CO_2_ on copper metabolism in the diatom *Thalassiosira pseudonana* (CCMP 1335), we employed an integrated eco-physiological, analytical chemistry, and transcriptomic approach to clarify the effect of increasing *p*CO_2_ on copper metabolism of *Thalassiosira pseudonana* across different temporal (short-term vs. long-term) and spatial (indoor laboratory experiments vs. outdoor mesocosms experiments) scales. We found that increasing *p*CO_2_ (1,000 and 2,000 μatm) promoted growth and photosynthesis, but decreased copper accumulation and alleviated its bio-toxicity to *T. pseudonana*. Transcriptomics results indicated that *T. pseudonana* altered the copper detoxification strategy under OA by decreasing copper uptake and enhancing copper-thiol complexation and copper efflux. Biochemical analysis further showed that the activities of the antioxidant enzymes glutathione peroxidase (GPX), catalase (CAT), and phytochelatin synthetase (PCS) were enhanced to mitigate oxidative damage of copper stress under elevated CO_2_. Our results provide a basis for a better understanding of the bioremediation capacity of marine primary producers, which may have profound effect on the security of seafood quality and marine ecosystem sustainability under further climate change.

## Introduction

Since the early 1900s, increasing atmospheric CO_2_ and associated ocean acidification (OA) have increased hydrogen ions (H^+^) concentration by 30% and dropped pH by 0.1 units in ocean surface waters (Vargas et al., 2017; Osborne et al., 2020). Unprecedented shifts in ocean chemistry are predicted to occur in the future, doubling the partial pressure of carbon dioxide and decreasing pH by 0.3 units by the end of the century (Shi et al., 2010; Gattuso et al., 2015). OA does not only alter the seawater pH but also changes the seawater carbonate system, including increased bicarbonate (HCO_3_^−^) and reduced carbonate (CO_3_^2−^) concentrations (Feely et al., 2004; Waldbusser and Salisbury, 2014). The shifts in seawater chemistry may alter the chemical behavior of other elements such as metals, modifying their bioavailability and thus toxicity (Millero et al., 2009; Roberts et al., 2013; Campbell et al., 2014; Bautista-Chamizo et al., 2016; Stockdale et al., 2016). Bautista-Chamizo et al. (2016) demonstrated that lower pH increased toxicity of zinc by altering the bioavailability to the marine microalgae *Pleurochrysis roscoffensis*. de Orte et al. (2014) also reported that OA enhanced the release of metals such as aluminum, iron, zinc, cobalt, lead and copper from sediments to the water column and thus increased their toxicity to *Phaeodactylum tricornutum*. In contrast, Shi et al. (2010) found that OA reduced the bioavailability of dissolved Fe and decreased the Fe uptake rate of diatoms and coccolithophores. Furthermore, the interactions between elevated CO_2_ and metals are species-specific, and dependent on species developmental stage, metal biochemistry and the degree of acidification (Ivanina and Sokolova, 2015). Previous studies investigating the effects of OA on phytoplankton have been predominantly based on short-term studies, and long-term investigations into the responses of marine phytoplankton to OA are limited. However, marine microbes have enormous potential for rapid adaptation to environmental changes, due to their large population sizes and short generation times (Thoms et al., 2012; Reusch and Boyd, 2013; Schlüter et al., 2014). Therefore, it is important to investigate responses of globally important microbes under evolutionary relevant timescales to obtain more accurate estimates of their resilience to environmental changes.

Copper (Cu) is an essential micronutrient for the metabolism of plants and algae, that is required for the functioning of proteins involved in photosynthesis (plastocyanin) and respiration (cytochrome *c*) and acts as a redox cofactor in many enzymes such as cytochrome *c* oxidase and copper/zinc superoxide dismutase (Andresen et al., 2018; Scheiber et al., 2019). However, the redox properties that make Cu an essential element also contribute to its inherent toxicity in excess concentrations and can induce oxidative stress and damage to macromolecules (Navarrete et al., 2019). To detoxify copper, algae and plants have evolved specific homeostatic mechanisms. One such detoxifying mechanism is the synthesis of metal-binding ligands, such as phytochelatins and metallothioneins and the subsequent distribution and compartmentalization of Cu within different cellular compartments. The acquisition of Cu from the environment depends on membrane transporter proteins such as the CTR-like Cu transporter, ZRT/IRT-like protein (ZIP), and cation diffusion facilitator (CDF). However, Cu needs to be reduced from Cu^2+^ to Cu^+^ by a plasma membrane ferric reductase (FRE) before import into the cell, which is expected to be the rate limiting process for Cu^2+^ transport in *Thalassiosira pseudonana*. Intracellular Cu^+^ may also be removed from the cell *via* Cu-transporting P_1B_-type ATPases (CTP) to reduce its toxicity (Guo et al., 2015; Huang et al., 2016; Liu et al., 2019; Zúñiga et al., 2020).

In line with OA, metal pollution is increasing in coastal environments due to increasing anthropogenic activities (for example, industrial, agricultural and domestic pollution) and this may affect the growth and species composition at the base of aquatic food webs (Davis et al., 2006; Miazek et al., 2015; Leung et al., 2017; Yung et al., 2017). Metals that form strong complexes with chloride (Cu^+^, Cd^2+^, and Hg^2+^) are mainly found in their free form and are not strongly influenced by changes in pH, while metals that form strong complexes with hydroxide (Al^3+^, Ga^3+^, In^3+^, and Be^2+^) or carbonate (Cu^2+^) will undergo significant changes in speciation as the pH of seawater decreases. It is expected that from 2,000 (pH 8.1) to 2,250 (pH 7.4) the fraction of Cu in the forms of CuCO_3_ and CuOH^+^ will decrease by 9 and 1%, respectively, while the toxic free ion concentration of copper (Cu^2+^) will increase by 24%, potentially increasing its bio-toxicity to marine biota (Millero et al., 2009).

Diatoms, as single-celled eukaryotes capable of photosynthesis, are distributed in marine and freshwater systems around the world (Armbrust et al., 2004), and are the most diverse algal group in the world, with at least 100,000 species (Falciatore et al., 2020). Diatoms contribute up to approximately 40% of oceanic primary productivity and are a critical component of coastal food webs, functionally sequestering carbon and nutrients and thereby playing a significant role in earth’s carbon cycle and the global biogeochemical cycles of nitrogen, phosphorus, and silicon (Armbrust, 2009; Wu et al., 2014; Benoiston et al., 2017). Early studies have indicated that diatoms respond differently to different global ocean changes. For example, it has been found that elevated CO_2_ enhances the growth rates of larger diatoms (Wu et al., 2014), and copper stress impacts diatoms at multiple cellular levels, including the morphological, behavioral, and physiological levels (Park et al., 2020). By conserving and utilizing energy in the cellular processes, diatoms have adopted unique adaptive strategies to respond to ocean warming and acidification (O'Donnell et al., 2018; Thangaraj and Sun, 2020; Zhong et al., 2021). Recent research has shown that OA reduced the toxicity of cadmium in *Phaeodactylum tricornutum* (Zhang et al., 2020). However, the metabolic pathways of copper in diatoms under future OA is unknown.

Here, we elucidated the effect of increasing CO_2_ on copper metabolism in the model diatom of *T. pseudonana*. Firstly, we characterized the effect of increasing CO_2_ on copper toxicity and its accumulation in *T. pseudonana* during a long-term selection period of 720 days. Secondly, to determine the adaptive capacity of *T*. *pseudonana* to increasing CO_2_, the long-term selected lines were grown under ambient or high CO_2_ with or without copper stress. Finally, we conducted the transcriptional and chemical analysis of long-term selected *T. pseudonana* under ambient and elevated *p*CO_2_ levels with or without copper exposure.

## Materials and methods

### Algal culture and experimental design

The diatom *T. pseudonana* (CCMP 1335) was obtained from the Yellow Sea Fisheries Research Institute Microalgae Culture Center of the National Marine Genetic Resource Center (Xu et al., 2022)^1^. In the laboratory, cells were grown in semi-continuous cultures in sterile seawater enriched with modified *f*/2 medium containing 100 μM N, 6 μM P and 100 μM Si and maintained at 20 ± 1°C under an irradiance of 120 μmol photons m^−2^ s^−1^ and a 12 h/12 h light–dark cycle (light on at 8:00 am and off at 8:00 pm).

### Determination the copper toxicity to the growth of *Thalassiosira pseudonana*

The growth inhibition of *T. pseudonana* under different concentrations of copper (Cu), was quantified before the start of the experiment. *T. pseudonana* was grown in 400 ml cultures at cell densities of 8 × 10^4^ cells ml^−1^ and supplemented with a series of copper (prepared with CuSO_4_·5H_2_O) ranging from 0 μmol L^−1^ (using the background concentration in natural seawater, < 0.03 μmol L^−1^) to 35 μmol L^−1^ for 96 h (during exponential phase). At the beginning and end of the experiment (96 h), 0.5 ml of *T. pseudonana* culture was collected and preserved in Lugol’s solution to estimate microalgal growth by directly counting cell numbers using a hemocytometer and optical microscope (Nikon, Tokyo, Japan). The growth rate (μ, day^−1^) was calculated using Equation (1):


(1) $$\mu =\left(\ln N_{1}-\ln N_{0}\right)/\left(t_{1}-t_{0}\right),$$

where N_1_ and N_0_ represent cell concentrations at t_1_ and t_0_, respectively. The percentage of growth inhibition was calculated with reference to the control (no additional copper) using Equation (2):


(2) $$percent\;of\;growth\;inhibition=\left(1-\mu_{2}/\mu_{1}\right)\times 100,$$

where μ_2_ and μ_1_ represent the growth rate at a certain copper concentration and in the absence of copper, respectively. The concentrations of copper causing a 50% reduction in growth (IC_50_, _96 h_, 20 μmol L^−1^, Supplementary Figure S1) was determined by linear interpolation. Although Cu was presented in the base medium, the group without extra copper addition was set as the control group. In the copper toxicity experiment, the compound EDTA (ethylenediaminetetraacetic acid, disodium salt, dehydrate; Na_2_EDTA·2H_2_O) and FeCl_3_·6H_2_O as well as other micronutrients in f/2 medium were not added into sterile seawater medium, due to the chelating properties of EDTA, which could decrease copper toxicity.

### Experimental design

Four experiments were set up in this study (Supplementary Figure S2). Firstly, to assess the effect of elevated *p*CO_2_ on the physiological performance of *T. pseudonana*, a selection experiment was set up in laboratory, where algae were cultured under ambient and elevated *p*CO_2_ for 720 days. Secondly, to assess the evolutionary response of *T. pseudonana* to elevated *p*CO_2_, shift experiments were set up in the laboratory, where the selected lineages at ambient and elevated *p*CO_2_ were transferred into five different concentrations of *p*CO_2_. Thirdly, to compare the differences in response between the indoor and outdoor experiments, an outdoor culture system was set up, where *T. pseudonana* was cultured under ambient and elevated *p*CO_2_ using natural temperature and light. Fourthly, we conducted transcriptome sequencing of *T. pseudonana* using the long-term selected population (after 720 days’ selection in laboratory), to gain a mechanistic understanding of how elevated *p*CO_2_ influences copper metabolism of *T. pseudonana*. For all four experiments, algae were acclimated under ambient or elevated *p*CO_2_ for more than 30 generations, and then exposed to Cu without acclimation. In all experiments, the respective *p*CO_2_ levels of 420, 1,000, and 2,000 μatm were established by bubbling the liquid medium with air or air/CO_2_ premixed gas using a CO_2_ chamber (HP1000G-D, China, for the laboratory experiment) or CO_2_ Enricher (CE-100B; Wuhan Ruihua Instrument & 25 Equipment Ltd., for the outdoor culture system).

### Selection experiment in laboratory

To study the effect of elevated CO_2_ on the physiological performance of *T. pseudonana*, three different *p*CO_2_ levels (420, 1,000, and 2,000 μatm; mimicking current and future *p*CO_2_ rises up to the year 2,300 under IPCC scenario RCP 8.5) were set up using nutrient-replete f/2 medium. *T. pseudonana* (acclimated at 20 ± 1°C under an irradiance of 120 μmol photons m^−2^ s^−1^) was transferred during the exponential phase of growth to an inoculum of the relevant CO_2_-modified f/2 medium at identical densities of 8 × 10^4^ cells ml^−1^ and the cultures diluted every 4–6 days to maintain a stable carbonate system. In the selection experiment, algal cells were grown under semi-continuous culture conditions under the above *p*CO_2_ levels for 720 days. Three biological replicates were set up using 1,000 ml flasks with 800 ml medium for each *p*CO_2_ treatment. To study whether long-term selection under elevated *p*CO_2_ has an impact on how *T. pseudonana* deal with exposure to copper stress, cells from each selected sample were transferred at a density of 8 × 10^4^ cells ml^−1^ into CO_2_-modified f/2 medium supplemented with or without copper for another 96 h at time intervals of 60 days (Supplementary Figure S2). Three concentrations of Cu exposure including a control (without additional Cu), low Cu (1 μmol L^−1^, as usually occurs in polluted coastal areas), and high Cu (20 μmol L^−1^, according to the IC_50_, _96 h_) were set up in the copper exposure experiment.

At the end of each batch-culture experiment, a 10 ml aliquot from each replicate was collected and centrifuged at 10,000 *g* for 10 min at room temperature. The algal precipitate was rinsed twice with Milli-Q water and an ice-cold phosphate buffer to remove extracellular copper, and then harvested and stored at −20°C to estimate intracellular copper content (Angel et al., 2017).

During the selection experiment the pH in each culture medium was measured before and after each dilution with a pH meter (Orion ROSS, Fisher Scientific Instruments), which was calibrated before use (NBS, National Bureau of Standards; variation range ± 0.05). Temperature, salinity, and total alkalinity (TA) were also measured periodically during the selection experiment at time intervals of 60 days. The average value was used to calculate carbonate system parameters using the CO2SYS Package (Pierrot et al., 2006; Supplementary Table S1).

### Shift experiment in laboratory

Following 720-day selection in the respective CO_2_ levels (corresponding to 833 generations, 920 generations and 887 generations in ambient *p*CO_2_ of 420 μatm and high *p*CO_2_ of 1,000 and 2,000 μatm, respectively), cells were transferred to another five increasing *p*CO_2_ concentrations, ranging from 420 to 2,000 μatm and maintained in semi-continuous batch culture and transferred to fresh media every 4–6 days (Supplementary Figure S2). After acclimation for 30 days, each lineage under the respective *p*CO_2_ was cultured with or without added exposure to Cu for another 96 h. The pH, temperature, salinity, and total alkalinity (TA) were measured before and after each dilution and the average was used to calculate the parameters of the carbonate system (Supplementary Table S2). Cells without Cu exposure were set as the control. In order to test acute Cu toxicity, high Cu (20 μmol L^−1^, according to the IC_50_, _96 h_) was used in the Cu exposure experiment. Cell growth and intracellular copper accumulation were determined after 96 h, using the methods described above. The direct response to the selection (S) was measured by comparing the growth rate (or intracellular copper concentration) of elevated *p*CO_2_ (1,000, 2,000 μatm) selected cells to that of cells from the ambient *p*CO_2_ culture using Equation (3) (Brennan et al., 2017)


(3) S=(E−A)/A,

where E is the growth rate (or intracellular copper concentration) of elevated *p*CO_2_ selected cells, and A is the growth rate (or intracellular copper concentration) of ambient *p*CO_2_ selected cells.

### Outdoor culture experiments

To investigate the effect of increasing *p*CO_2_ on copper metabolism of *T. pseudonana* under more natural environmental conditions, cells selected at *p*CO_2_ values of 420, 1,000, and 2,000 μatm for 720 days in laboratory were transferred outdoors and cultured at the respective *p*CO_2_ on the sea (37°06 ‘N, 122°33 ‘E), using 10 L tanks with 8 L cultures, which were set as long-term selection treatments and deployed on the sea surface (LT; Supplementary Figure S3). Additionally, stock cells without any selection treatment in the laboratory were also transferred to outdoors and cultured under the same series of *p*CO_2_ of 420, 1,000, and 2,000 μatm, and set as short-term acute treatments and cultures also deployed on the sea surface (ST). After acclimation in filtered natural seawater under the respective *p*CO_2_ for 30 days, each lineage was cultured with or without additional Cu exposure for another 96 h. Cell growth and intracellular copper accumulation were determined after 96 h, using the methods described above. The variations of pH, temperature, salinity, and total alkalinity (TA) were measured every day, and were used to calculate the parameters of the carbonate system (Supplementary Figure S4).

### Transcriptome analysis and enzyme activity determination

To investigate the molecular responses of *T. pseudonana* to increasing *p*CO_2_ and copper exposure, cultures evolved under 420 and 1,000 μatm *p*CO_2_ for 720 days were transferred in triplicate to fresh media under the same CO_2_ conditions, with or without copper exposure (20 μmol L^−1^) for a further 24 h. At the end of the experiment, 250 ml of culture from each replicate were harvested, centrifuged at 4°C and frozen in liquid nitrogen and stored at −80°C for RNA extraction, transcriptional sequencing, and RT-qPCR. The primers designed for reference and target genes were designed and are listed in Supplementary Table S3. The raw sequence data reported in this paper have been deposited in the Genome Sequence Archive in BIG Data Center, Beijing Institute of Genomics, Chinese Academy of Sciences, under accession numbers CRA001653, that are publicly accessible at http://bigd.big.ac.cn/gsa. A further 100 ml of culture from each replicate was harvested, centrifuged at 4°C and frozen in liquid nitrogen and stored at −80°C for enzyme activity determination. The antioxidant enzyme activities of glutathione peroxidase (GPX), catalase (CAT) and ascorbate peroxidase (APX) were determined using commercial reagent kits (Comin Biotechnology Co., Ltd., Suzhou, China) following the manufacturer’s instructions.

### Statistical analysis

The effects of elevated CO_2_ and Cu exposure on algal growth rate or intracellular copper concentration ([Cu]_intra_) over the selection period of 720 days were analyzed with a mixed effects model in R,^2^ using the packages lme4 and lmerTest. Algal growth rate or intracellular copper concentration ([Cu]_intra_) were used as the experimental variable to be explained. Replicates were used as random effects. To test the individual and combined effects of increasing *p*CO_2_ and Cu exposure on algal growth rate and intracellular copper concentration [Cu]_intra_ across different culture duration in selection experiment, different *p*CO_2_ levels or different Cu exposure scenario or culture duration was set as one fixed effect. Different *p*CO_2_ levels, different Cu exposure scenario, and culture duration were then tested separately and each in interaction with *p*CO_2_, and the most parsimonious model was chosen for reporting based on the smallest AICc score (Akaike Information Criterion for small sample sizes); To test the algal adaptive response to increasing *p*CO_2_ in shift experiment, the assay *p*CO_2_ was set as a fixed factor and the different selection *p*CO_2_ was used as random effects; To test algal physiological responses to increasing *p*CO_2_ in outdoor culture experiment, different *p*CO_2_ levels were used as fixed factors. The different Cu exposure scenarios and selection length (short-term, STS and long-term, LTS) were used as random effects.

## Results

### Elevated *p*CO_2_ increased growth rate and reduced intracellular copper accumulation of *Thalassiosira pseudonana* in the laboratory selection experiment

**Figure 1:**
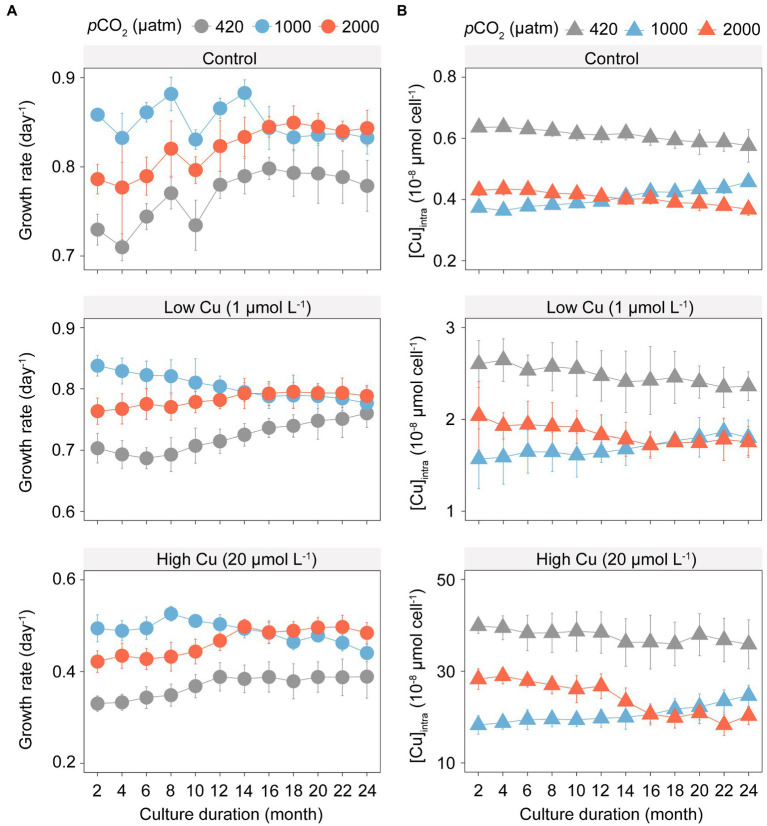
Growth rates and intracellular copper concentration of *T. pseudonana* in the selection experiment under ambient (420 μatm, grey) and elevated *p*CO_2_ (1,000 μatm, blue; and 2,000 μatm, red) with three gradients copper exposure. **(A)** Changes in growth rate (day^−1^); **(B)** Shift in intracellular copper concentration ([Cu]_intra_). The symbols represent the means and error bars represent standard deviations of triplicate cultures.

Elevated *p*CO_2_ significantly increased growth rates of *T. pseudonana* in all Cu exposure scenarios (Figure 1A; *F*_4,88_ = 4.00, *p* < 0.05). Under elevated *p*CO_2_ conditions without Cu, maximal growth rates were found at 1000 μatm *p*CO_2_ until month 14 (Figure 1A; *F*_2,92_ = 159.71, *p* < 0.001). Trajectories of algal growth rate over the course of the selection experiment differed substantially between the ambient and elevated *p*CO_2_. After acclimation for 24 months (approximately 1,000 generations), populations selected under 2,000 μatm *p*CO_2_ showed higher growth rates than populations selected at 1,000 and 420 μatm *p*CO_2_. In addition, the variation in growth rates between the ambient and elevated *p*CO_2_ reduced as the experiment proceeded (*F*_2,100_ = 56.03, *p* < 0.001).

In contrast to the variation in growth rate, elevated *p*CO_2_ significantly reduced copper accumulation within algal cells in all Cu exposure scenarios (Figure 1B; *F* = 79.632, *p* < 0.001). Populations selected under 1,000 and 2,000 μatm *p*CO_2_ showed opposite trends in copper accumulation across the selection periods. Over 24 months of selection, copper accumulation gradually increased in populations selected under 1,000 μatm *p*CO_2_, whereas copper accumulation gradually decreased in populations selected under 2,000 μatm *p*CO_2_, in each of the control, the low Cu or high Cu treatments.

### Evolution under elevated CO_2_ increases population growth rates in shift experiment

**Figure 2:**
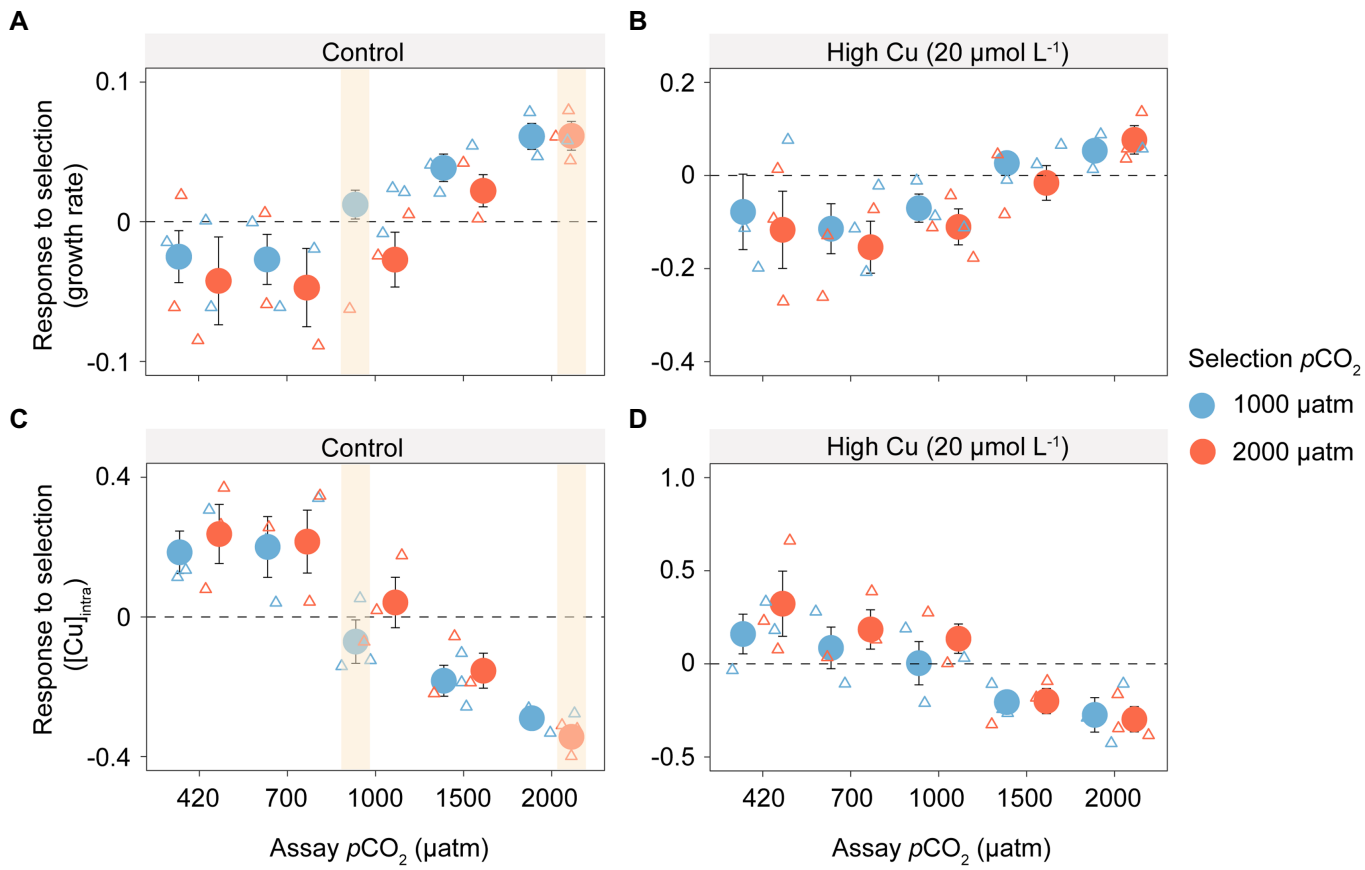
Growth rates and intracellular copper concentration of *T. pseudonana* in the shift experiment under elevated *p*CO_2_ with or without additive copper exposure. Box plots show the response to selection measured as the difference in growth rate (day^−1^) without **(A)** or with **(B)** copper exposure and intracellular copper concentration ([Cu]_intro_) without **(C)** or with **(D)** copper exposure, between populations evolved to elevated CO_2_ (1,000 and 2,000 μatm) and the evolved control populations (420 μatm), under the same environmental conditions. The direct response to selection is highlighted in yellow and compares the population growth rate of a population evolved in the selection environment with the plastic response of a control population to that same environment. The dashed line shows the average growth rate of the evolved control populations in each environment (indicated on x axis and panel labels). Distance from the dashed line shows the difference in responses between the evolved populations and the evolved control in the same environment.

We found evidence of adaptation to elevated CO_2_ conditions by measuring the direct response to selection (growth rate of evolved populations relative to that of control assayed in the same selection environment). Populations evolved under 1,000 μatm and 2,000 μatm *p*CO_2_ showed a positive response to selection in their respective selection environments, indicating that evolution under elevated *p*CO_2_ conditions increases growth rates beyond the plastic response (of the evolved control populations). While populations evolved under elevated *p*CO_2_ conditions converge on a similar evolved growth rates (Figure 1), populations evolved under the highest *p*CO_2_ conditions (2,000 μatm) evolved more than populations evolved under *p*CO_2_ 1,000 μatm to arrive at the same endpoint. However, the strength of selection in each environment is similar (indicated by the initial population growth rate of evolved control populations in the same environments; Figure 2). Population growth rates generally increased with increasing *p*CO_2_ across all selected lines, including treatments without (Figure 2A; *F*_4,38_ = 6.441, *p* < 0.001) or with Cu exposure (Figure 2B; *F*_4,38_ = 2.924, *p* < 0.05). However, growth rates in elevated *p*CO_2_-selected samples decreased and showed lower levels than the ambient-selected ones, when they were shifted back to ambient *p*CO_2_ at 420 μatm or a slightly higher *p*CO_2_ at 700 μatm.

When copper is added to the environments, simulating environmental pollution of the aquatic environment, the benefits of evolution under elevated *p*CO_2_ are reversed in all but the highest *p*CO_2_ environments (Figures 2B,D; copper accumulation is greater in selected lines than the plastic response indicated by the dashed line). Overall, copper accumulation decreased significantly with increasing *p*CO_2_ in the shift experiments under treatments both without (Figure 2C; *F*_4,40_ = 11.23, *p* < 0.001) and with Cu exposure (Figure 2D; *F*_4,40_ = 5.12, *p* < 0.001), indicating that copper accumulation may be reduced in photosynthetic algae under future *p*CO_2_ conditions.

### The effect of increasing *p*CO_2_ on growth rate and intracellular copper accumulation of *Thalassiosira pseudonana* in the outdoor culture experiments

**Figure 3:**
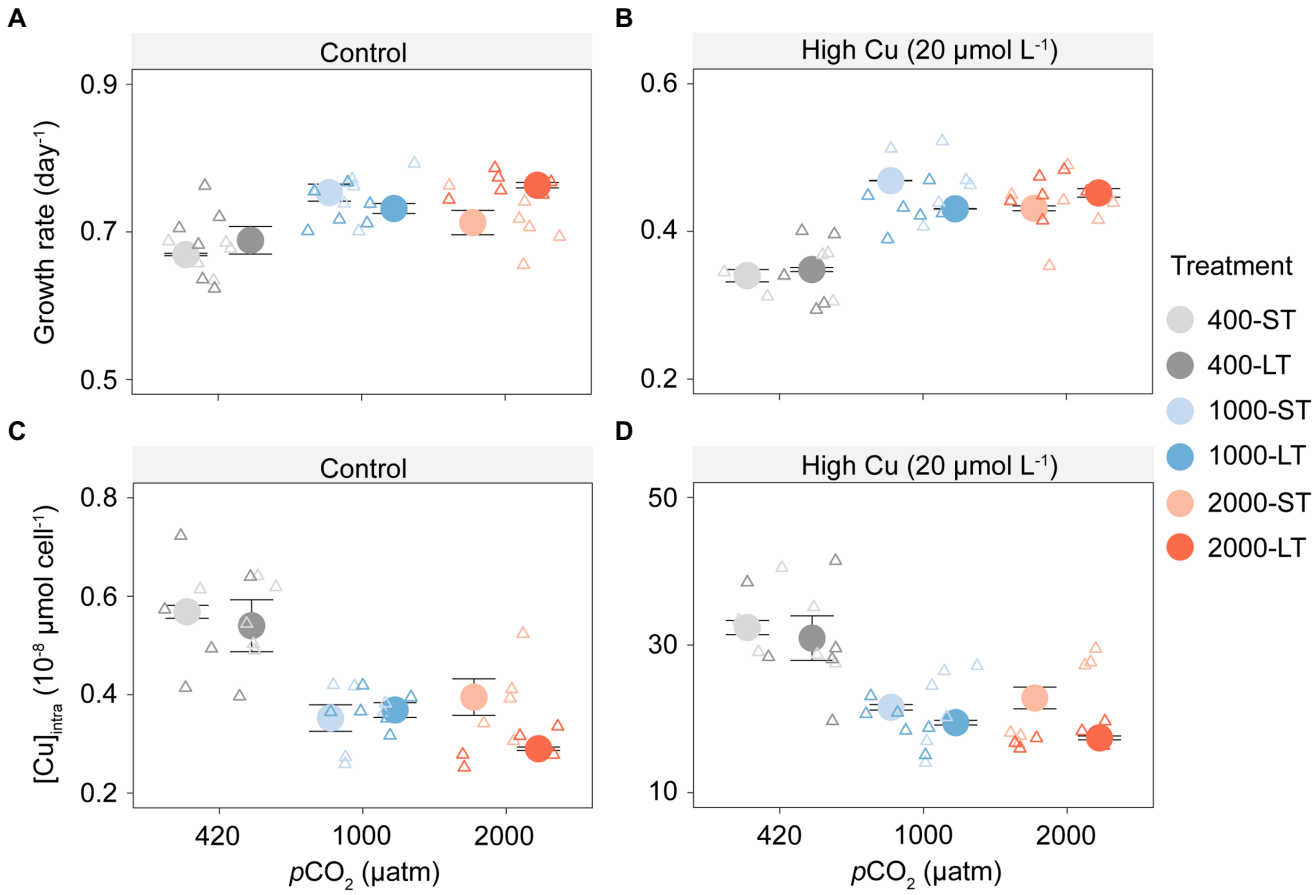
Growth rates and intracellular copper concentration of *T. pseudonana* in outdoor culture experiments after short-term (ST) and long-term (LT) acclimation under different *p*CO_2_ with or without added copper. Changes in growth rate to increasing *p*CO_2_ without **(A)** or with **(B)** copper exposure; Shift in intracellular copper concentration ([Cu]_intra_) to increasing *p*CO_2_ without **(C)** or with **(D)** copper exposure. The values represent the means and error bars represent standard deviations of triplicate cultures.

In outdoor culture experiments, population growth rates increased under elevated *p*CO_2_ (1,000 and 2,000 μatm), in both the short-term (ST) or long-term (LT) acclimation experiments and in all copper treatments (Figures 3A,B; *F*_1,69_ = 28.40, *p* < 0.001). By comparison between ST and LT samples, the former showed the highest growth at 1,000 μatm *p*CO_2_, whereas the latter showed a maximum at 2,000 μatm *p*CO_2_.

Elevated *p*CO_2_ (1,000 and 2,000 μatm) also reduced copper accumulation across all experimental treatments, even using the natural seawater where the background Cu concentration was less than 0.03 μmol L^−1^ (Figures 3C,D; *F*_1,68_ = 15.18, *p* < 0.001). Copper accumulation in the short-and long-term mirrored the pattern found for growth rates. Under the control treatment without Cu exposure, intracellular copper concentration ([Cu]_intra_) in ST samples was reduced by 38.0 and 30.5% under 1,000 and 2,000 μatm *p*CO_2_ respectively, and that in LT samples was reduced by 31.7 and 46.3% under 1,000 and 2,000 μatm *p*CO_2_, respectively. Under Cu exposure, [Cu]_intra_ in ST samples was reduced by 33.4 and 29.5% under 1,000 and 2,000 μatm, respectively, and that in LT samples was reduced by 37.0 and 43.7% under 1,000 and 2,000 μatm *p*CO_2_, respectively_._

### Variation of gene expression and enzyme activity of *Thalassiosira pseudonana* under elevated *p*CO_2_ with or without copper exposure

**Figure 4:**
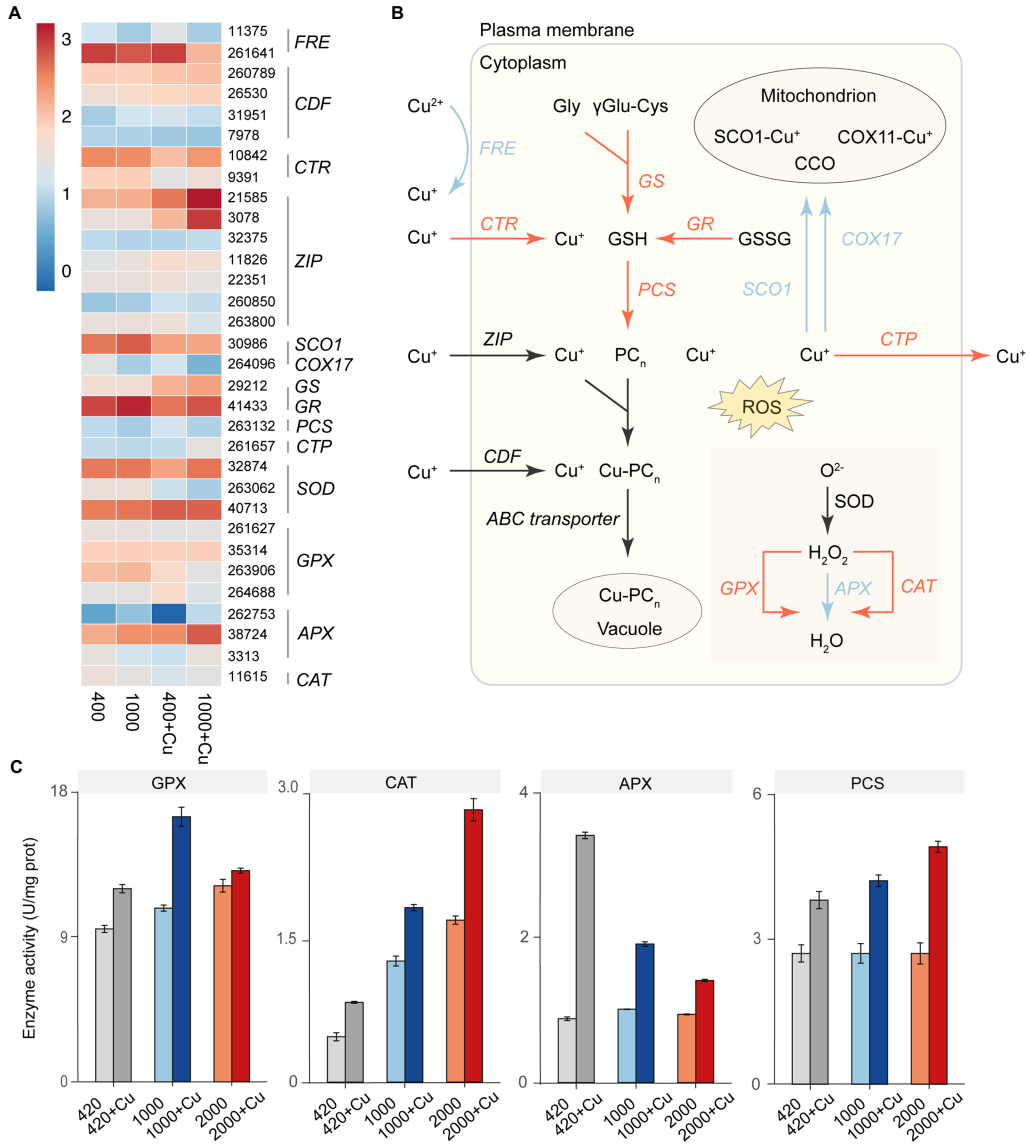
The effect of elevated *p*CO_2_ on genes expression and enzyme activity involved in the copper metabolic pathway of *T. pseudonana*. **(A)** Heat map of representative gene expression in copper metabolic pathway of *T. pseudonana* under elevated *p*CO_2_ with or without copper exposure. **(B)** Schematic of the altered copper metabolic pathway in *T. pseudonana* under elevated *p*CO_2_. Arrows in red or blue represented the up-or down-regulated genes under elevated *p*CO_2_. Black arrows indicated that there were not consistent variation trends for the functionally similar genes with different ID number. **(C)** Activities of three antioxidant enzymes (units/mg protein), including, glutathione peroxidase (GPX), ascorbate peroxidase (APX), and catalase (CAT) under elevated *p*CO_2_ with or without copper exposure.

Transcriptome analysis as well as real-time quantitative polymerase chain reaction (RT-qPCR; Figure 4A; Supplementary Figure S5) revealed 32 differentially expressed genes (Supplementary Dataset 1) associated with copper metabolism among the different treatments. Although the Cu^+^ transporter *CTR* was up-regulated under elevated *p*CO_2_, the reduction reaction from Cu^2+^ to Cu^+^ by *FRE* was significantly down-regulated. As a result, Cu^2+^ uptake rate was reduced (Supplementary Figure S6). Within the cell, glutathione synthetase (*GS*), glutathione reductase (*GR*), and phytochelatin synthetase (*PCS*) were up-regulated to synthesize phytochelatins (PC_n_), which can be used to chelate with Cu^+^ and sequester it in the vacuole. Elevated *p*CO_2_ also resulted in down-regulation of the gene expression of Cu chaperones (*COX17* and *SCO1*) to decrease Cu toxicity to mitochondrion. Cu-transporting P1B-type ATPases (*CTP*) were up-regulated under elevated *p*CO_2_ to enhance the efflux of intracellular Cu^+^ from the cell, this was also verified by a measured increase in Cu efflux rate under elevated *p*CO_2_ (Supplementary Figure S7). Additionally, to eliminate reactive oxygen species (ROS) induced by Cu stress under elevated *p*CO_2_, glutathione peroxidase (GPX) and catalase (CAT) were up-regulated both in gene expression and enzyme activity (Figure 4C). However, ascorbate peroxidase (APX) showed decreased activity under higher *p*CO_2_ (1,000 and 2,000 μatm), compared with ambient *p*CO_2_ (420 μatm), when exposed to copper.

## Discussion

In this study, we provided both a quantitative and a mechanistic understanding of how diatoms might respond to heavy metal stress under future ocean acidification using both a 720-day laboratory selection experiment, outdoor culture experiments, and transcriptomic sequencing. Long-term selection experiments showed that elevated *p*CO_2_ promoted algal growth rate and reduced copper accumulation within algal cells, and thus alleviated copper stress to *T. pseudonana*. Shift experiments using the long-term selected cells indicated adaptive evolution potential of *T. pseudonana* under elevated *p*CO_2_. Biochemical and transcriptomic analysis further showed that *T. pseudonana* employed a specific copper detoxification strategy under elevated *p*CO_2_.

### Adaptive potential of long-term selected *Thalassiosira pseudonana* under elevated *p*CO_2_

Many studies have found different responses between short-term acclimation and long-term adaptation of phytoplankton to elevated *p*CO_2_ (Schlüter et al., 2016; Li et al., 2017; Tong et al., 2018). Our findings are in line with previous studies indicating selection under elevated *p*CO_2_ promoted algal growth rates in diatom species (Wu et al., 2014; Zhu et al., 2017; Qu et al., 2018). The positive effect of elevated *p*CO_2_ may result from the reduced energy requirement of carbon concentration mechanisms (CCMs) with the saved energy being used to support carbon fixation and growth (Raven et al., 2011). Hopkinson et al. (2011) reported that doubling of ambient *p*CO_2_ reduced CCM-related energy expenditure by ~20% and decreased the total energy demand on carbon fixation by up to 6%. As the selection experiment continued, the response differences between ambient and elevated *p*CO_2_ decreased among different treatments after 24 months selection, which may be due to an effect induced by adaptation to the laboratory setting (Collins, 2016). Shift experiments further confirmed the evolutionary responses of *T. pseudonana* to high *p*CO_2_, in that the long-term selected samples under elevated *p*CO_2_ showed a lower growth rate than ambient selected ones when they were transplanted back into ambient *p*CO_2_ conditions. Collins and Bell (2004) found that the long-term selected cells under high *p*CO_2_ showed less efficient carbon concentration mechanisms, or a higher per-cell requirement for inorganic carbon, which could result in decreased growth rate when they were assayed under ambient *p*CO_2_.

### Elevated *p*CO_2_ reduces cooper toxicity to *Thalassiosira pseudonana*

When the algae were exposed to copper stress, elevated *p*CO_2_ significantly reduced intracellular copper accumulation and alleviated the negative effect of copper stress (Supplementary Figure S8). Changes to the seawater carbonate chemistry under elevated *p*CO_2_ did not change external copper concentration in the culture medium (Supplementary Figure S9), suggesting that reduced copper accumulation under elevated *p*CO_2_ is biologically mediated. This is supported by the study of de los Santos et al. (2019) who also found that projected OA would ameliorate copper toxicity on photosynthetic performance of *Zostera noltei* when pH decreased from 8.36 to 8.03. Our findings add to the growing body of evidence that adaptive evolution of the marine diatom community under projected OA would increase their resilience to harsh environments (Domingues et al., 2014; Valenzuela et al., 2018; Dong et al., 2020; Xu et al., 2022).

Previous studies have documented that elevated *p*CO_2_ does not only directly affect primary producers but also changes the distribution, speciation and bioavailability of organic and inorganic trace metals and will therefore modify their interaction with organisms (Millero et al., 2009; Campbell et al., 2014; Stockdale et al., 2016). Decreasing pH could increase the concentration of Cu^2+^ by reducing complex formation with CO32-and OH^−^, thereby altering the bioavailability and increasing copper toxicity to marine organisms (Millero et al., 2009; Roberts et al., 2013). On the other hand, there is competition between H^+^ and Cu to prevent Cu^2+^ from binding at the cell surface (Gao et al., 2017). The decreased inhibitory effect of Cu on *Ulva prolifera* under elevated *p*CO_2_ (1,000 μatm) suggests that the competition between H^+^ and Cu outcompetes the increased availability of Cu^2+^ in the medium (Gao et al., 2017). Here, although the effect of increasing *p*CO_2_ on Cu speciation in the medium were not determined, the free Cu^2+^ concentration should increase in background seawater medium as Millero et al. (2009) has previously indicated. However, results consistently found reduced Cu bioaccumulation in *T. pseudonana* under elevated *p*CO_2_, both with and without additional Cu exposure (Figures 1–3). Therefore, it suggested that the alleviation of toxicity could be due to the elevated CO_2_ *per se* but not to the reduced pH.

### Altered detoxification strategy employed by *Thalassiosira pseudonana* to cope with copper toxicity under elevated *p*CO_2_

Transcriptome analysis indicated that algal selected under elevated *p*CO_2_ performed a specific copper detoxification strategy, that includes down-regulation of the reduction reaction from Cu^2+^ to Cu^+^ at the cell membrane to decrease copper uptake, up-regulation of biosynthesis of phytochelatins to transform free toxic Cu^+^ to less toxic organic forms, and up-regulation of a Cu^+^ transporter to enhance Cu^+^ efflux from the cell to decrease the concentration of free ions of Cu in the algal cells. All these processes could mitigate the oxidative stress in cells and enhance its tolerance to Cu exposure (Figure 4). Recent studies have demonstrated that Cu^2+^ is reduced to Cu^+^ extracellularly, which is an obligatory first step in Cu uptake in an oceanic diatom and is mediated by biological processes (Kong and Price, 2020).

Our results indicated that 1 and 20 μΜ Cu exposure inhibited growth and induced significant oxidative stress and damage in *T. pseudonana* (Figures 1, 4). Although Cu is one of the redox trace elements in biological systems and a basic cofactor of many enzymes (Wang and Ki, 2019), excess copper induces oxidative stress in the diatom and increases accumulation of reactive oxygen species (ROS), which would destroy macromolecules such as proteins, nucleic acids and lipids (Anu et al., 2016). In response to the oxidative stress resulting from copper exposure, *Chlamydomonas reinhardtii* was found to increase activities of the antioxidant enzymes glutathione S-transferase (GST), glutathione peroxidase (GPX), superoxide dismutase (SOD) and peroxidase (POD) to eliminate ROS (Zheng et al., 2011; Jiang et al., 2016). Bielmyer-Frasera et al. (2018) studied the physiological responses of two coral species, *Acropora cervicornis* and *Pocillopora damicornis* to OA and copper exposure. They found that copper exposure increased activities of the antioxidant enzymes CAT, GPX, and GR of these two species. By comparison with copper exposure alone, copper exposure under high *p*CO_2_ (1,000 μatm) further increased activities of CAT, GPX, GR of *Acropora cervicornis.* This is supported by our results in that the high *p*CO_2_ selected *T. pseudonana* showed higher activities of GPX and CAT when exposed to external stress than ambient-grown cells did.

## Conclusion

Our results indicate that elevated *p*CO_2_ promotes growth and decreases Cu accumulation in a diatom, and the response to OA depends on the *p*CO_2_ level and the timescale of OA, which sheds new light on how carbon enrichment might counteract the negative effects of copper toxicity. However, whether the influence of elevated *p*CO_2_ on Cu bio-toxicity to *T. pseudonana* is consistent with other microorganisms and with other trace metals or other environmental pressures remains to be further investigated. Furthermore, transcriptomic analysis demonstrated that the long-term selected diatoms under elevated *p*CO_2_ employed a specific copper detoxification strategy under further OA scenarios, indicating phenotypic trait responses to OA resulting from genetic influences. This highlights the importance of long-term selection on the potential of algae to adapt to elevated *p*CO_2_ and thus change their biotic response to abiotic environmental changes. This detoxification effect could be transmitted from a primary producer through trophic transfer in the food chain, and may help us to understand the resilience potential of marine primary producers and maintain fisheries and ecosystem security under global climate change. Altogether, our study provides novel insights on the biogeochemical cycle of copper regulated by marine primary producers under global climate change.

## Data availability statement

The datasets presented in this study can be found in online repositories. The names of the repository/repositories and accession number(s) can be found in the article/Supplementary material.

## Author contributions

NY designed the study. DX and SH provided the environmental and ecological context. DX, SH, XF, XZ, WW, JB, GB, and NY analyzed the data and interpreted the results. DX, SH, JB, GB, and NY wrote the manuscript. All authors contributed substantially to manuscript revisions.

## Funding

This work was supported by the National Natural Science Foundation of China (41976110 and 31772075); the Young Taishan Scholars Program to DX, Taishan Scholars Funding and Talent Projects of Distinguished Scientific Scholars in Agriculture; Marine S&T Fund of Shandong Province for Pilot National Laboratory for Marine Science and Technology (Qingdao) (Nos. 2021QNLM050103-1); Central Public-interest Scientific Institution Basal Research Fund, CAFS (NO. 2020TD27); and China Agriculture Research System (CARS-50).

## Conflict of interest

The authors declare that the research was conducted in the absence of any commercial or financial relationships that could be construed as a potential conflict of interest.

## Publisher’s note

All claims expressed in this article are solely those of the authors and do not necessarily represent those of their affiliated organizations, or those of the publisher, the editors and the reviewers. Any product that may be evaluated in this article, or claim that may be made by its manufacturer, is not guaranteed or endorsed by the publisher.

## Footnotes

^1^ http://marine.fishinfo.cn/

^2^ https://www.r-project.org/
