# Supplementary material for: Elevated CO2 reduces copper accumulation and toxicity in the diatom Thalassiosira pseudonana
Source: Front Microbiol. 2023 Jan 6;13:1113388. doi: 10.3389/fmicb.2022.1113388 (PMC9853397; doi:10.3389/fmicb.2022.1113388)
Supplement: Supplementary file 2 [file Data_Sheet_1.PDF]

Supplementary information for

Elevated CO_2 reduces copper accumulation and toxicity in the diatom *Thalassiosira pseudonana*

Dong Xu[a,b,#], Shujie Huang[a,#], Xiao Fan[a], Xiaowen Zhang[a], Yitao Wang[a], Wei Wang[a], John Beardall[c], Georgina Brennan[d,*], Naihao Ye[a,b,*]

[a]Yellow Sea Fisheries Research Institute, Chinese Academy of Fishery Sciences, Qingdao, China

[b]Function Laboratory for Marine Fisheries Science and Food Production Processes, Qingdao National Laboratory for Marine Science and Technology, Qingdao, China

[c]School of Biological Sciences, Monash University, Clayton, Victoria 3800, Australia

[d]Institute of Marine Sciences, ICM-CSIC; Barcelona, Spain

[#] These authors contributed equally to this work.

[*]Corresponding author: Naihao Ye, Yellow Sea Fisheries Research Institute, Chinese Academy of Fishery Sciences, Qingdao, China. Email: yenh@ysfri.ac.cn; Georgina Brennan, Molecular Ecology and Fisheries Genetics Laboratory, School of Biological Sciences, Bangor University, Bangor LL57 2UW, UK. Email: g.l.b.doonan@gmail.com

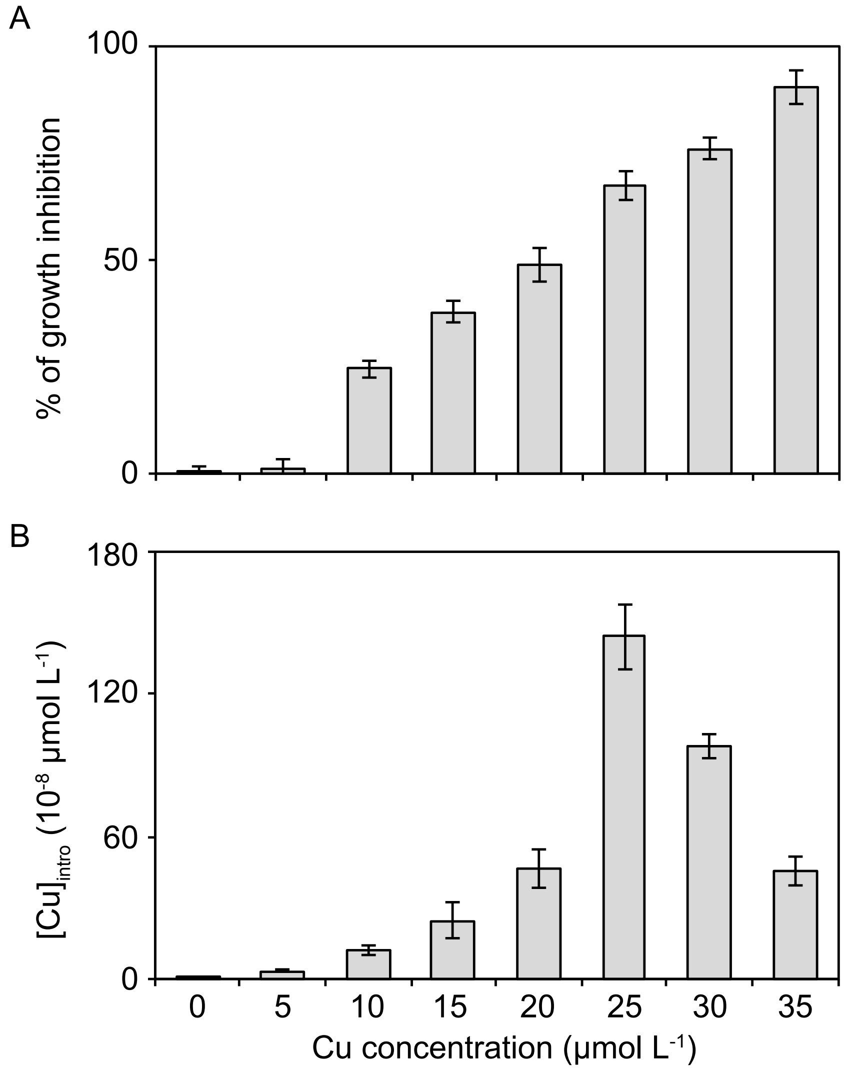

Figure S1. The inhibiting effect of copper on growth and copper accumulation of *T. pseudonana.* (A) Effect of different total copper concentrations on the percentage of growth inhibition after 4 days. (B) Copper accumulation by *T. pseudonana* with different copper treatment concentrations. The data show that the concentration of copper leading to 50% inhibition of growth of *T. pseudonana* is 20 μM after copper exposure for 4 days.

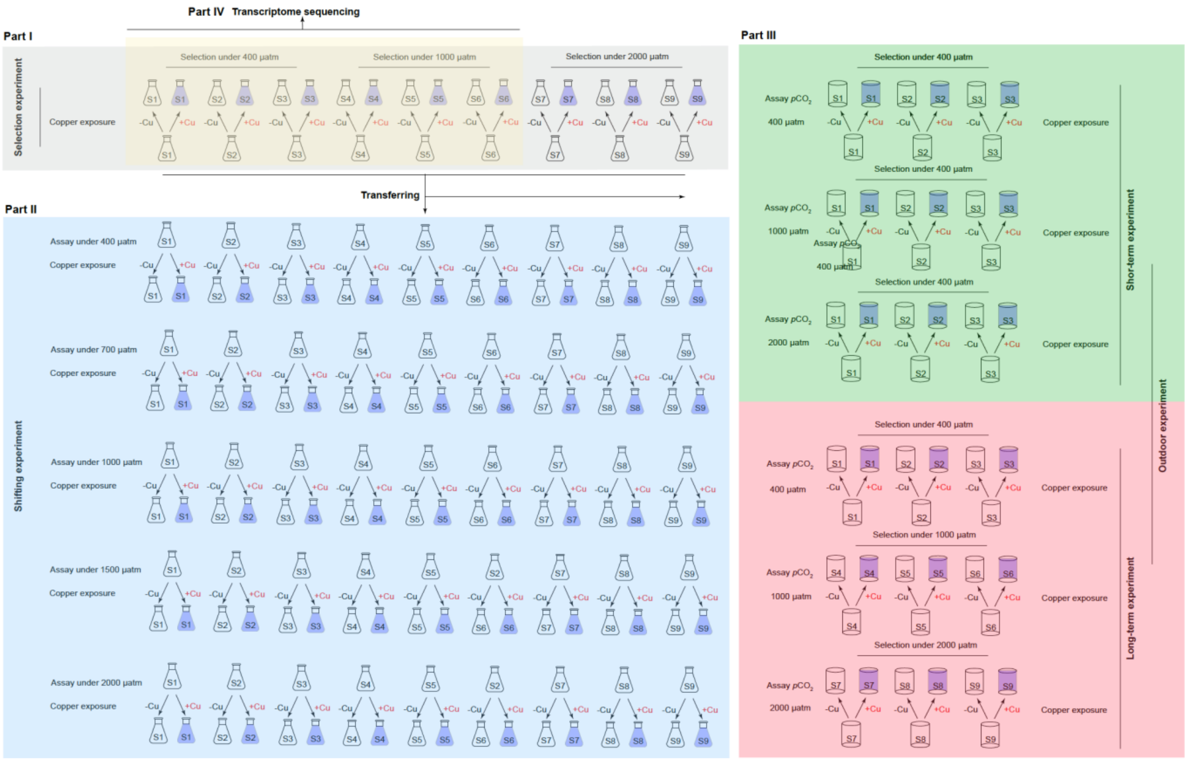

Figure S2. A schematic diagram of the experimental set up in the study. Firstly, to assess the effect of elevated $p\mathrm{CO_2}$ on physiological performance of *T. pseudonana*, a selection experiment was set up in laboratory, where algae were cultured under ambient and elevated $p\mathrm{CO_2}$ for 720 days. Secondly, to assess the evolutionary response of *T. pseudonana* to elevated $p\mathrm{CO_2}$, shift experiments were set up in laboratory, where the selected lineages at ambient and elevated $p\mathrm{CO_2}$ were transferred into another five concentrations of $p\mathrm{CO_2}$. Thirdly, to compare the differences in response between the indoor and outdoor experiments, an outdoor culture system was set up, where *T. pseudonana* was cultured under ambient and elevated $p\mathrm{CO_2}$ using natural temperature and light. Fourthly, we conducted transcriptome sequencing of $p\mathrm{CO_2}$ using long-term selected populations (after 720 days' selection in the laboratory), to gain a mechanistic understanding of how elevated $p\mathrm{CO_2}$ influences copper metabolism of *T. pseudonana*. For all four parts of experiments, algae were acclimated under ambient or elevated $p\mathrm{CO_2}$ (depending on the selection environment), and then exposed to Cu without acclimation.

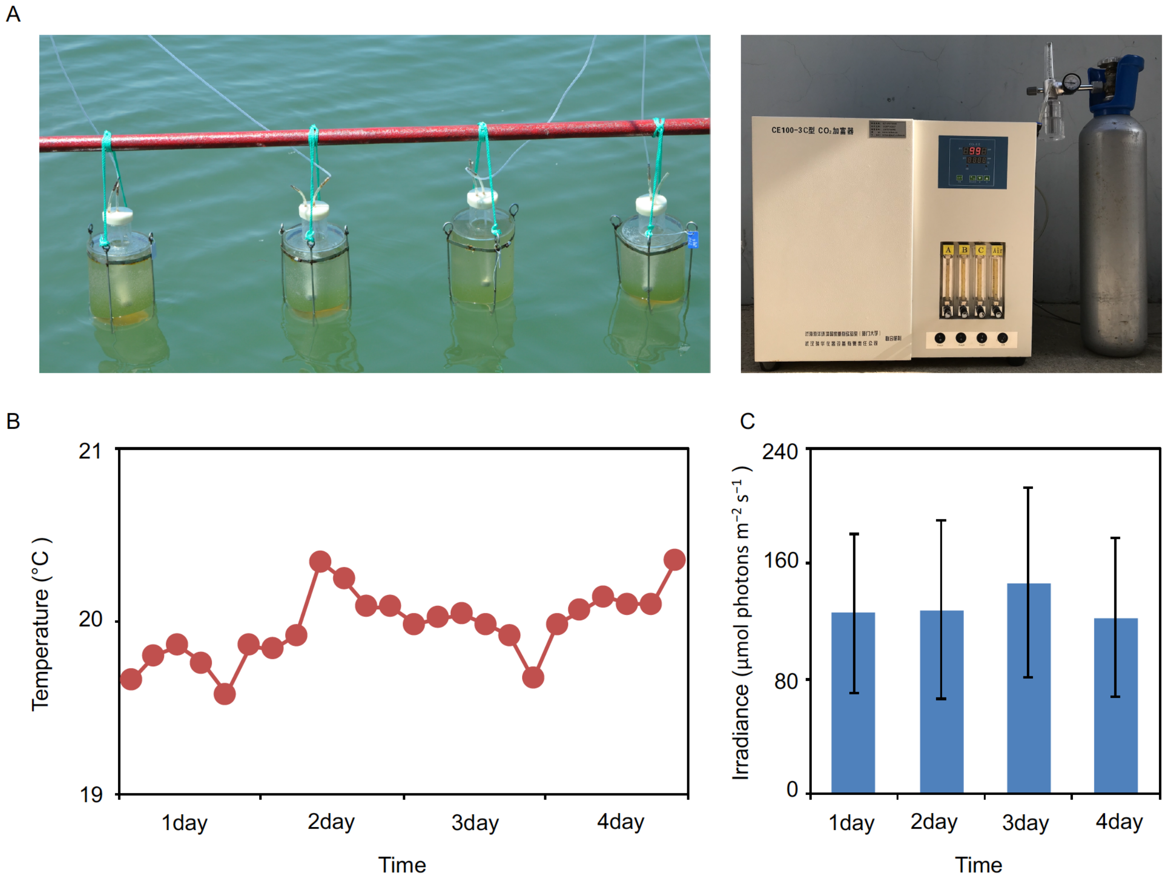

Figure S3. Outdoor culture setup. (A) A photograph of the mesocosm setup. (B) Variation of seawater temperature over the culture period of 4 days. (C) Variation of mean daily irradiance at the seawater surface over the culture period of 4 days. Low and high pCO$_2$ levels were controlled by bubbling the mesocosm tanks with air or mixed gas using a CO$_2$ Enricher. During the culture period, the seawater temperature (mean ± standard errors) fluctuated slowly from 19.70± 0.03°C to 20.20 ± 0.06 °C. The experimental design followed that of the laboratory experiment, with three replicate tanks per treatment (i.e. n=3 per treatment).

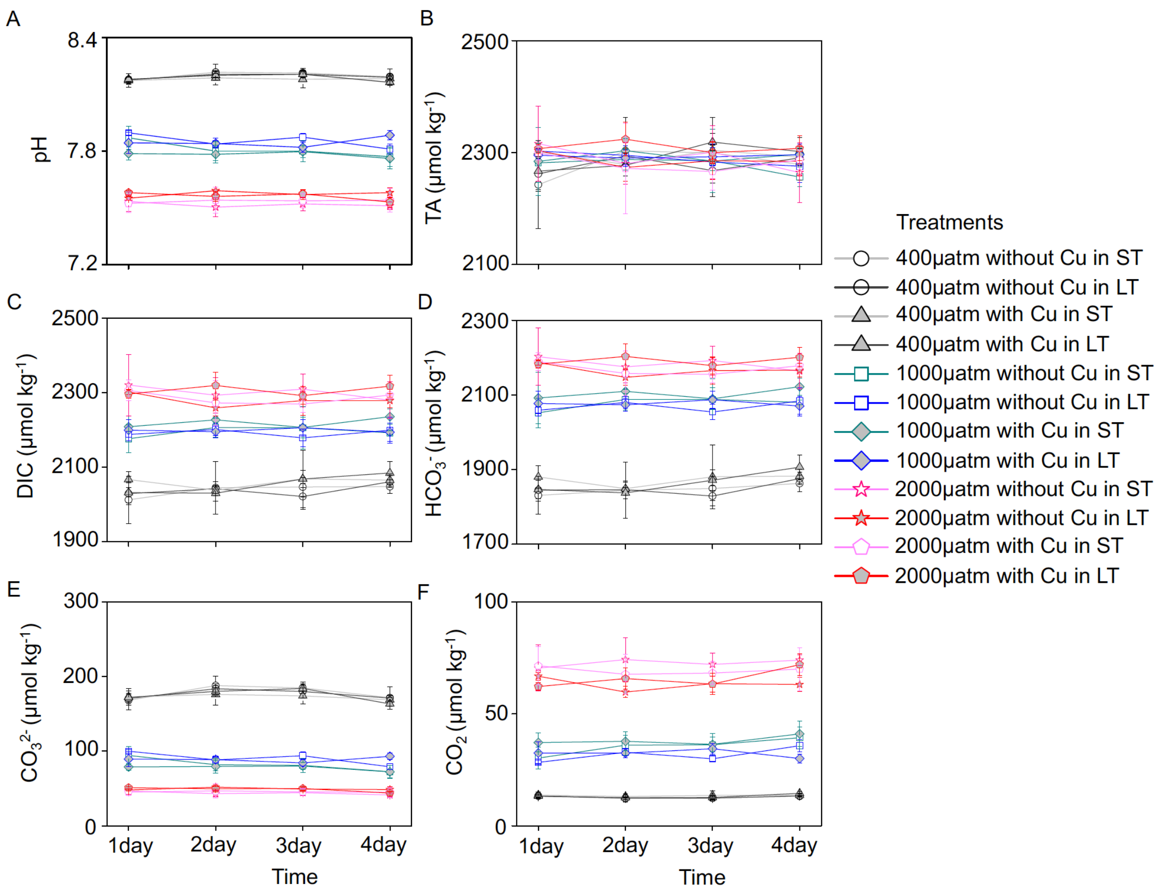

Figure S4. Variation of seawater carbonate chemistry parameters (mean ± standard errors) under low or high *p*CO_2 levels over a time period of 4 days in the outdoor culture experiments. (A) pH, (B) TA, (C) DIC, (D) HCO_3^-, (E) CO_3^{2-}, (F) CO_2. Increased *p*CO_2 significantly altered the carbonate chemistry in the outdoor culture, but the carbonate chemistry was stable over the duration of the experiment.

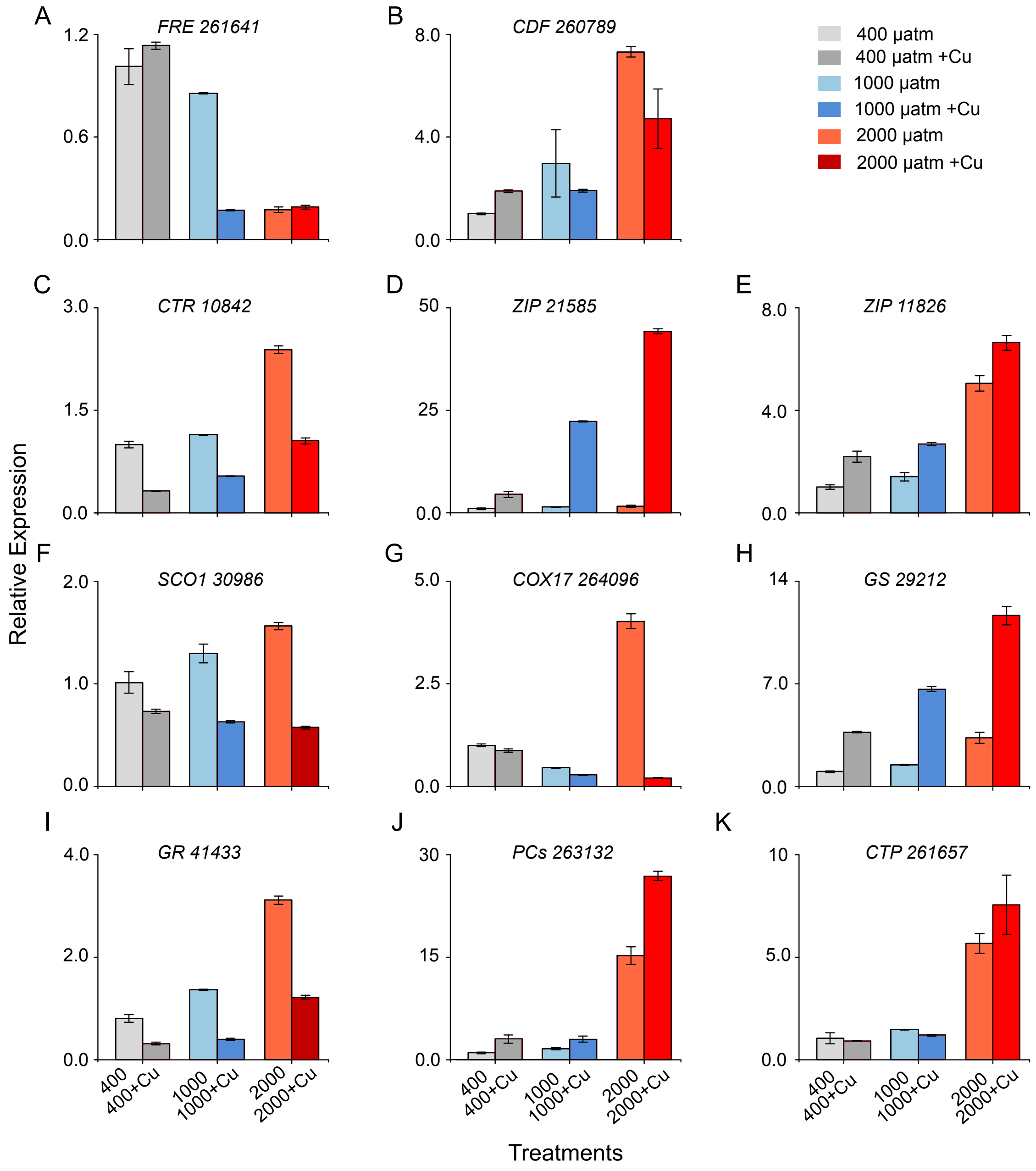

Figure S5. The relative expression of genes involved in the copper metabolic pathway of *T. pseudonana* as demonstrated by RT-qPCR under increasing *p*CO_2 with or without additional copper exposure.

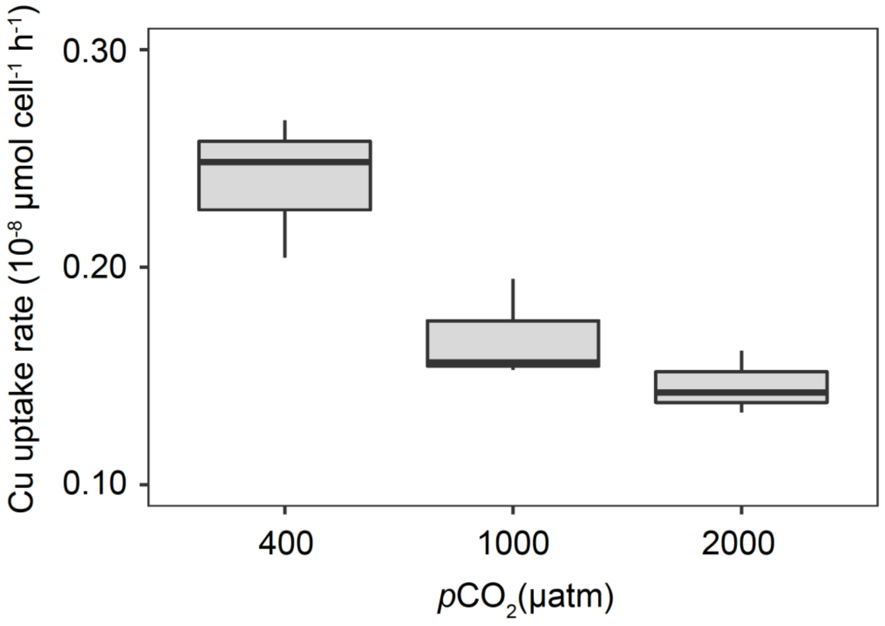

Figure S6. The effect of increasing *p*CO_2 on the Cu uptake rate of *T. pseudonana* over 12 h in short-term emergency experiments under copper exposure. The data show that elevated *p*CO_2 significantly reduced the copper uptake rate of *T. pseudonana* ($P<0.05$).

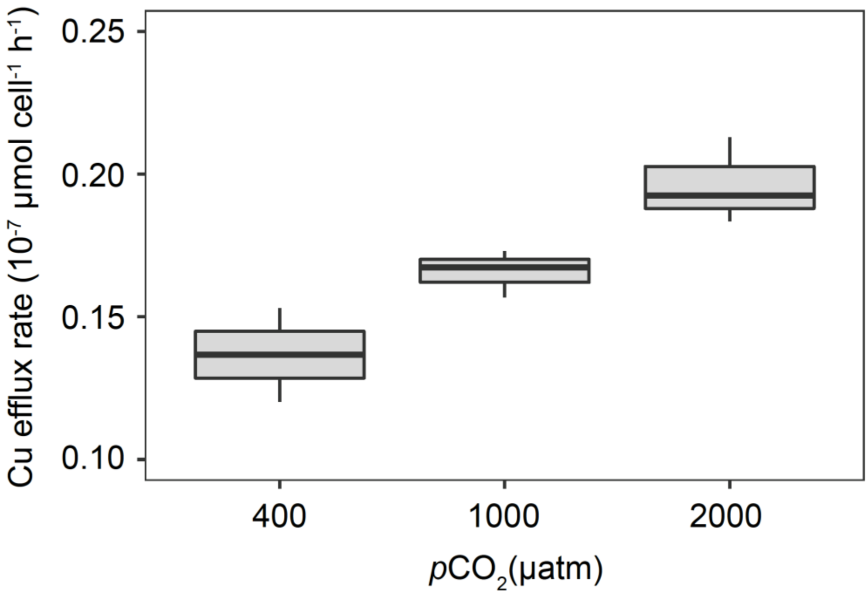

Figure S7. The effect of increasing *p*CO_2 on the Cu efflux rate of *T. pseudonana* pre-exposed to Cu for 96 h. The data show that elevated *p*CO_2 significantly enhanced the copper efflux rate of *T. pseudonana* ($P<0.05$).

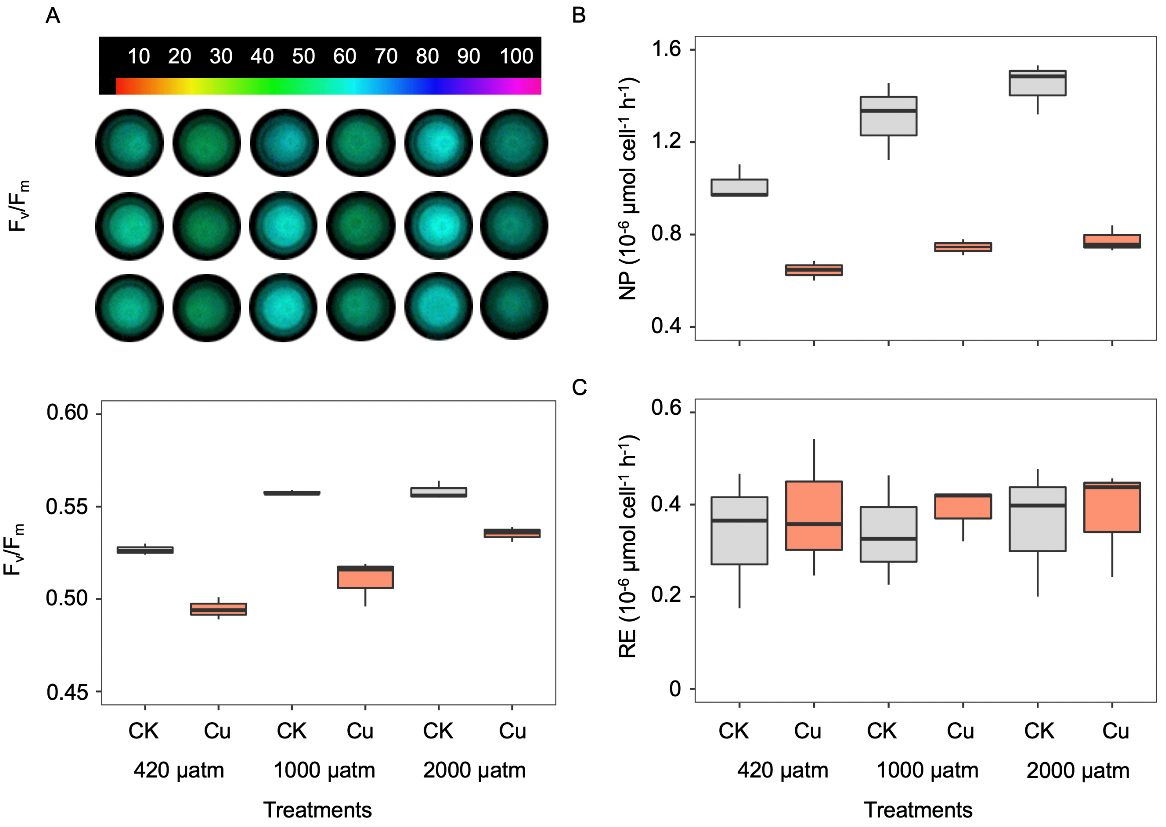

Figure S8. Physiological performance associated with photosynthesis and respiration of *T. pseudonana* after long-term selection in laboratory studies with or without added copper exposure. (A) Variation of images representing maximum quantum yield (F_v/F_m) of photosystem II under different pCO_2 after acclimation for 720 days of *T. pseudonana.* (B) The effect of increasing pCO_2 on net photosynthetic rate (NP) after acclimation for 720 days of *T. pseudonana*. (C) The effect of increasing pCO_2 on respiratory rate (RE) after acclimation for 720 days in laboratory. Each data point is an average of three biological replicates and error bars represent standard deviation (n=3).

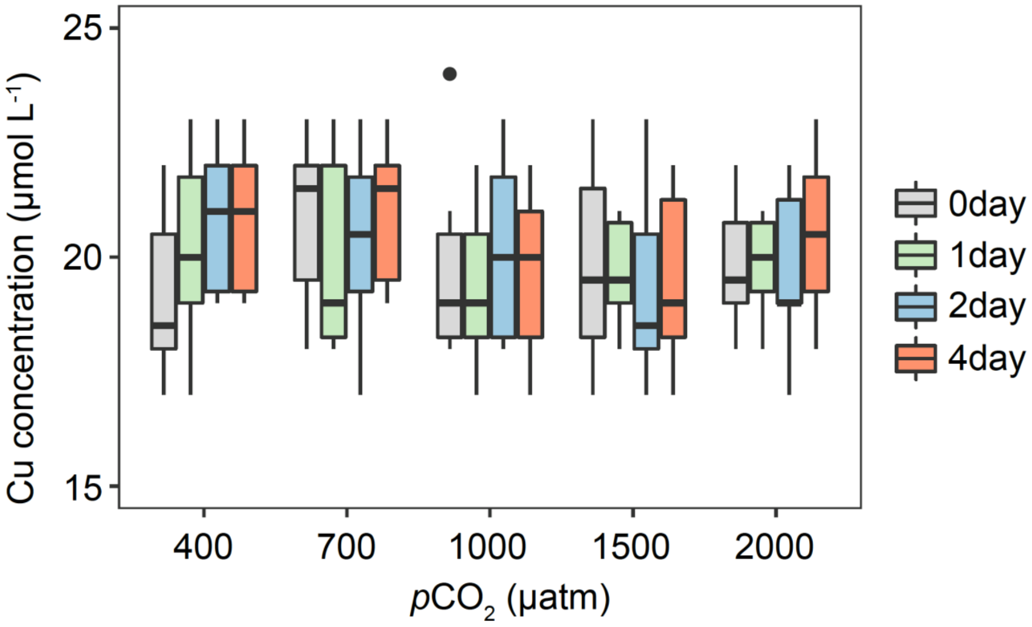

Figure S9. The effect of elevated CO_2 on the copper concentration in seawater for 4 days. Colored columns show the observed copper concentration of three biological replicates (± SE) under elevated pCO_2 from 400 µatm, 700 µatm, 1000 µatm, 1500 µatm, to 2000 µatm. It demonstrates that there were not any significant impacts of increasing pCO_2 on copper concentration in seawater.

Table S1. The average value (mean ± standard errors) of carbonate system parameters in the selection experiment using the CO2SYS Package.

pCO_2 (μatm)		pH	TA (μmol L^{-1})	DIC (μmol L^{-1})	HCO_3^- (μmol L^{-1})	CO_3^{2-} (μmol L^{-1})	CO_2 (μmol L^{-1})
400	CK	8.20±0.02	2323±62	2068±57	1869±52	186±8	1.26±1
	Low Cu	8.20±0.01	2352±24	2098±20	1897±17	187±4	12.9±1
	High Cu	8.19±0.01	2329±57	2080±57	1883±55	183±5	13.0±1
1000	CK	7.86±0.04	2375±28	2269±19	2139±17	98±10	32±3
	Low Cu	7.77±0.03	2393±4	2318±15	2195±17	82±5	40±3
	High Cu	7.78±0.03	2358±27	2280±22	2160±20	81±5	39±2
2000	CK	7.66±0.04	2372±39	2334±38	2218±36	64±6	53±6
	Low Cu	7.57±0.05	2369±28	2361±10	2242±11	52±7	66±8
	High Cu	7.50±0.01	2362±35	2374±35	2253±33	45±2	76±2

Table S2. The average value (mean ± standard errors) of the carbonate system in the shift experiment using the CO2SYS Package.

pCO_2 (μatm)		pH	TA (μmol L^{-1})	DIC (μmol L^{-1})	HCO_3^- (μmol L^{-1})	CO_3^{2-} (μmol L^{-1})	CO_2 (μmol L^{-1})
400	Without Cu	8.19±0.01	2329±46	2083±50	1889±50	181±1	13±1
	With Cu	8.15±0.01	2337±38	2112±40	1928±40	168±3	15±1
700	Without Cu	7.92±0.03	2344±36	2215±41	2079±43	109±6	27±2
	With Cu	7.90±0.01	2320±42	2200±41	2069±38	103±3	28±1
1000	Without Cu	7.81±0.01	2270±35	2186±38	2069±37	83±1	35±2
	With Cu	7.78±0.04	2374±35	2297±28	2176±25	82±8	39±3
1500	Without Cu	7.67±0.03	2367±44	2327±52	2211±50	65±2	51±4
	With Cu	7.72±0.02	2362±44	2305±43	2188±41	72±4	45±2
2000	Without Cu	7.53±0.04	2347±47	2350±34	2231±34	48±5	71±6
	With Cu	7.55±0.04	2348±63	2347±67	2229±63	49±4	69±7

Table S3. The primers designed for quantitative RT-PCR in *T. pseudonana* (CCMP1335).

Gene ID	Gene function	Abbreviation	Primers (5′- 3′)
Thaps3-269504	actin-related protein centractin-like protein	*CK*	F:TCTGTATGCCTCGGGACG
			R:CTCTACCAGCCAAATCACTCCT
Thaps3-261641	Ferric reductase like protein	*FRE*	F: TCGACGATATGGCTCACTCG
			R: TGCACGAGGCATTACCCAAA
Thaps3-260789	Cation efflux protein	*CDF*	F: TCGAGAGTGTCGGTTCGTTG
			R: CCAAGTAGGCACCTCCACAA
Thaps3-10842	CTR copper transporter	*CTR*	F: TGGCAACTCTGCTGACCATT
			R: TCTACCCAAGTTCCCTCCGT
Thaps3-21585	ZIP family transporter	*ZIP*	F: TAGTCATACCCGCCATGCAC
			R: CTTCTGCATGCTTCCCCTCA
Thaps3-11826	ZIP family transporter	*ZIP*	F: TCTCTGTTCGACGGACTTGC
			R: CCAAGCAAGATGGACCCCAA
Thaps3-30986	Copper chaperone	*SCO1*	F: CTTTGGTTTCGCTCGGTGTC
			R: TGGCATAGTCGCGTAATGCT
Thaps3-264096	Cytochrome C oxidase copper chaperone	*COX17*	F: AAAGTGGGCATCAAATCAGGC
			R: CAACCTCTTACAATCCGCGT
Thaps3-29212	Glutathione synthase	*GS*	F: AGAACGGCAACATGTCGGAT
			R: GAGCTTGGACTTGCAAACCG
Thaps3-41433	Glutathione reductase	*GR*	F: TGGGATGCACCAAAGCAGAT
			R: CACAACCGACTCACCAGTCA
Thaps-263132	Phytochelatin synthase-like protein	*PCS*	F: GGATGTGACGGAATGGTCGT
			R: TGGCACTTGACGAGATTGCT
Thaps3-261657	Heavy metal P1B-ATPases	*CTP*	F: GTACCCTTACTCACGGACGC
			R: GCCGATACAAGAGTCGCAGA
